# Genome assembly and annotation of a *Drosophila simulans* strain from Madagascar

**DOI:** 10.1111/1755-0998.12297

**Published:** 2014-07-14

**Authors:** NICOLA PALMIERI, VIOLA NOLTE, JUN CHEN, CHRISTIAN SCHLÖTTERER

**Affiliations:** Institut für Populationsgenetik, Vetmeduni ViennaVeterinärplatz 1, 1210, Wien, Austria

**Keywords:** alternative splicing, *de novo* assembly, *Drosophila simulans*, genome annotation, pseudogenes, RNA-Seq, transcriptome reconstruction

## Abstract

*Drosophila simulans* is a close relative of the genetic model *D. melanogaster*. Its worldwide distribution in combination with the absence of segregating chromosomal inversions makes this species an increasingly attractive model to study the molecular signatures of adaptation in natural and experimental populations. In an effort to improve the genomic resources for *D. simulans*, we assembled and annotated the genome of a strain originating from Madagascar (M252), the ancestral range of *D. simulans*. The comparison of the M252 genome to other available *D. simulans* assemblies confirmed its high quality, but also highlighted genomic regions that are difficult to assemble with NGS data. The annotation of M252 provides a clear improvement with alternative splicing for 52% of the multiple-exon genes, UTRs for 70% of the genes, 225 novel genes and 781 pseudogenes being reported. We anticipate that the M252 genome will be a valuable resource for many research questions.

## Introduction

*Drosophila simulans* is a close relative of the model organism *Drosophila melanogaster*, from which it diverged 2–8 million years ago (Obbard *et al*. 2012). Like its sister species, it shows a widespread geographical distribution and has adapted to a large variety of environments. Despite that the two species are phenotypically very similar and share the same habitat, they differ in a number of traits related to behaviour, development, morphology and tolerance to environmental stresses [for a review, see Capy & Gibert (2004)]. At the molecular level, *D. simulans* populations were frequently found to be more polymorphic than *D. melanogaster* and showed higher levels of linkage disequilibrium (i.e. Hamblin & Veuille 1999; Rozas *et al*. (2001), but see Nolte *et al*. (2013)). *D. melanogaster* and *D. simulans* have a compact (∼140 Mb) and highly collinear genome, with the exception of a large fixed paracentric inversion on chromosome arm 3R (Sturtevant & Plunkett 1926) and a few small inversions on the other chromosomes [reviewed in (Horton 1939; Aulard *et al*. 2004)]. In contrast to *D. melanogaster*, *D. simulans* has very few segregating inversions (Aulard *et al*. 2004), which provides an advantage for the analysis of natural variation: the suppression of recombination caused by segregating inversions in *D. melanogaster* not only complicates the interpretation of polymorphism patterns in natural populations (Corbett-Detig & Hartl 2012; Kapun *et al*. 2014), but also leads to many false positives in evolve and resequence studies (i.e. Tobler *et al*. (2013)). Thus, it has been proposed that *D. simulans* could be better suited for experimental evolution studies compared *D. melanogaster* (Tobler *et al*. 2013).

The first published *D. simulans* genome was obtained from a mixture of strains that were sequenced at low coverage (Begun *et al*. 2007), and it is currently being maintained at FlyBase (http://flybase.org). While this assembly is of very low quality due to its shallow coverage, recently one of the strains, the North American strain w501, has been resequenced at deeper coverage and higher quality (Hu *et al*. 2012). For the other strains, only the data for the initial low coverage sequencing (∼1x) are available (http://www.genomesonline.org). Given the increasing importance of *D. simulans* for evolutionary studies, we generated a new *de novo* assembly of an African strain from Madagascar (strain M252), which represents the ancestral species range (Kopp *et al*. 2006). We compared our new African genome to the existing *D. simulans* genomes and found it to be of similar quality as the w501 genome (Hu *et al*. 2012). Importantly, we also provide an extensive annotation of protein-coding genes and pseudogenes. Unlike the annotation of previous *D. simulans* genomes, our annotation integrates orthology alignments with RNA-Seq data from several developmental stages. This new, extended annotation provides a substantial improvement by including novel isoforms, UTRs and novel genes.

## Materials and methods

### Samples and libraries

We generated two paired-end libraries from *D. simulans* adult female specimens of the M252 strain collected in 1998 by B. Ballard in Madagascar and provided by D. J. Begun. We will refer to the libraries as DNA-Seq data set, used for the genome assembly and RNA-Seq data set, used for the annotation:

1. DNA-Seq data set: We extracted genomic DNA from 43 adult females of strain M252 using a high salt extraction protocol (Miller *et al*. 1988) and fragmented it on a Covaris S2 device (Covaris, Inc. Woburn, MA, USA). The library was constructed using the NEBNext DNA Library Prep Master Mix Set (New England Biolabs, Ipswitch, MA, USA) for end repair, A-tailing and ligation with standard Illumina paired-end adapters. Size selection on an agarose gel was followed by PCR amplification for 10 cycles with Phusion polymerase (NEB) and paired-end primers PE 1.0 and PE 2.0 (Illumina, San Diego, CA, USA). The library was sequenced at BGI (Hong Kong) on Illumina HiSeq 2000. Read length is 100 bp, and fragment size (including the reads) ranges between 400 and 680 bp.
2. RNA-Seq data set: we generated pooled mRNA from multiple developmental stages: first, second, third instar larvae, early, intermediate and late pupal stage, adult virgin males and females (2 h old), adult mated males and females (several days old). For each developmental stage and category of adults, we pooled several individuals and extracted total RNA independently from each pool using a standard TriFast protocol (Peqlab, Erlangen, Germany). Total RNA was quantified using the Qubit Fluorometer (Invitrogen, Carlsbad, CA, USA), and equal quantities from each pool were combined for library construction. Poly-A selection followed the Illumina protocol for paired-end mRNA libraries. The mRNA was fragmented in 1× first strand buffer for 1 min at 94°C and reverse-transcribed using random primers and SuperScript II Reverse Transcriptase (Invitrogen). After purification on Agencourt RNAClean XP beads (Beckman Coulter, Brea, CA, USA), second strand synthesis was carried out with dUTP replacing dTTP. The library was constructed as described for the DNA-Seq data set, but PCR-amplified for 12 cycles. The library was sequenced at BGI (Hong Kong) on Illumina HiSeq 2000 using a strand-specific protocol. Read length is 100 bp, and fragment size (including the reads) ranges between 210 and 350 bp.

### *De novo* assembly

The M252 genome was assembled following the protocol described in Nolte *et al*. (2013). In brief, paired-end reads from the DNA-Seq data set were trimmed using the script trim_fastq.pl from PoPoolation (Kofler *et al*. 2011) with quality threshold 18 and a minimum read length of 50 bp. Trimming statistics are reported in Table S1 (Supporting information). The *de novo* assembly was generated from a subset of 50 million paired-end reads using CLC Genomics Workbench 5.0.1. Anchoring of the resulting scaffolds (by default, CLC connects contigs by gaps with a size estimated from spanning paired-end reads) on the *D. melanogaster* genome release 5.22 was performed with the nucmer module from the MUMmer package v.3.0 (Delcher *et al*. 2002) (parameters -mum -c 30 -g 1000 -b 1000 -l 15) (Kurtz *et al*. 2004). Using the show-tiling module from the MUMmer (Delcher *et al*. 2002) package, scaffolds were arranged into super-scaffolds as described in Nolte *et al*. (2013).

### Assembly comparison

We compared our assembly to the references from FlyBase release 1.4 and from Hu *et al*. (2012) version 2 (http://genomics.princeton.edu/AndolfattoLab/w501_genome.html). To obtain those genomic regions shared among the three assemblies, we aligned the genomes with Mauve 2.3.1 and extracted the largest common syntenic region from chromosome arms 2L, 2R, 3L and 3R. Percentage of Ns was calculated for these truncated references. Assembly quality was assessed using an independent African *D. simulans* sample derived from a pool of 50 isofemale lines containing a total of 42 655 534 reads (Illumina pipeline 1.4 – paired-ends – 2 × 100 bp – insert-size 450 bp) (Nolte *et al*. 2013). Reads were trimmed using trim_fastq.pl from PoPoolation (Kofler *et al*. 2011) (parameters –quality-threshold 20 –fastq-type sanger –min-length 50 –no-5p-trim) and mapped with DistMap (Pandey & Schlötterer 2013) using the BWA 0.5.8c (parameters -o 1 -n 0.01 -l 200 -e 12 -d 12) to the FlyBase reference release 1.4, to the assembly from Hu *et al*. (2012) and to the *de novo* assembly of M252. We used BWA in combination with the specified mapping parameters as it had been previously shown to perform well for Pool-Seq data (Kofler *et al*. 2011). Assembly quality was evaluated by computing average coverage and average percentage of nonproper pairs for each chromosome and for 10 kb sliding windows using, respectively, the scripts calculate-coverage+GC.pl and broken-pairs.pl from Nolte *et al*. (2013) (http://www.popoolation.at/mauritiana_genome/index.html). Nonproper pairs were defined as reads belonging to one of the following categories: (i) one of the mates is unmapped, (ii) one of the mates is mapped to a different chromosome/contigs, (iii) one of the mates is mapped to the same strand as the other mate, (iv) the distance between the mates is outside the expected range.

### Orthology annotation

**Fig 1:**
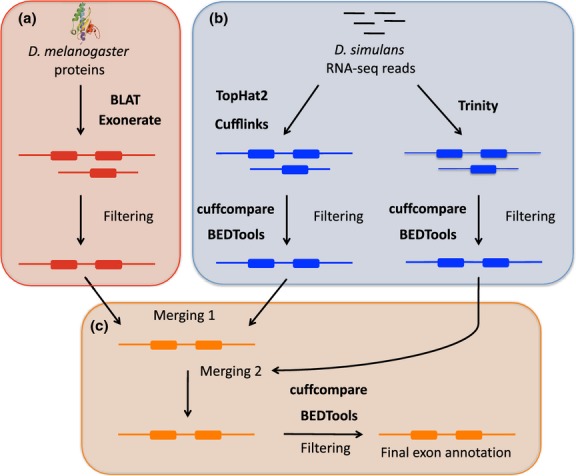
Schematic summary of the annotation pipeline. For details, refer to the text. a) Orthology annotation, b) RNA-Seq annotation, c) Merging annotation tracks.

A schematic summary of the whole annotation pipeline is reported in Fig. 1a. For the orthology annotation, *D. melanogaster* proteins from FlyBase (release 5.49) were aligned to our assembly using Exonerate version 2.2.0 (Slater & Birney 2005) (parameters –model protein2genome –bestn 1 –showtargetgff -q) and BLAT (Kent 2002) (parameters –maxIntron = 50 000). Gene models obtained from Exonerate were subjected to the following filters: (i) proteins with more than one best hit were excluded, (ii) proteins with internal frameshifts or premature stop codons were removed and separately retained for the identification of pseudogenes, (iii) proteins with <95% of aligned query length were excluded, (iv) isoforms with identical intron-exon structure were collapsed into a single isoform. These transcripts correspond to *D. melanogaster* isoforms of the same gene sharing the coding sequence (CDS) but having different UTRs, (v) proteins coming from different *D. melanogaster* genes aligned to the same genomic location were excluded, (vi) gene models with less than 95% total sequence overlap between Exonerate and BLAT were removed, (vii) gene models with no BLAT alignment were removed if they had a different number of exons than the corresponding *D. melanogaster* orthologue. Genes removed at step 2 were further classified as pseudogenes if their exonerate and BLAT gene models had at least 95% total length overlap.

### RNA-Seq annotation

Reads from the RNA-Seq data set were initially processed using two different pipelines (Fig. 1B), to generate two independent annotations that will be later merged (see the section Merging annotation tracks): (i) TopHat/Cufflinks and (ii) Trinity. In pipeline (i), reads from the RNA-Seq data set were trimmed using the fastq-trim.pl script from PoPoolation (Kofler *et al*. 2011) (parameters –quality-threshold 20 –fastq-type illumina –min-length 40; Table S1, Supporting information) and mapped with TopHat2 (Kim *et al*. 2013) (parameters –phred64-quals -p 4 -r 200) to the M252 assembly. Alignments were filtered for properly paired-reads using samtools 0.1.18. As high coverage can confound transcript reconstruction (Palmieri *et al*. 2012), a subset of randomly sampled 50M read pairs was used for transcriptome reconstruction with Cufflinks (Trapnell *et al*. 2010) version 2.0.2 (default parameters). In pipeline (ii), the trimmed reads were processed with Trinity (Grabherr *et al*. 2011) read normalization (parameters –max_cov 30 –pairs_together –SS_lib_type RF) and assembled using the Trinity (Grabherr *et al*. 2011) *de novo* transcriptome assembly module (parameters –SS_lib_type RF). Transcripts obtained by Trinity were aligned to the M252 genome using BLAT (parameters –minIdentity = 98 –maxIntron = 50000). A single best unambiguous hit for each transcript was selected if the aligned length of both query and target sequences was larger than 99%. Finally, Cufflinks (Trapnell *et al*. 2010) with the -G option was used for both Cufflinks and Trinity annotations to compute transcript-based expression in RPKM (Reads Per Kilobase of Million mapped reads) and transcripts with RPKM <1 where removed.

### Merging annotation tracks

At first, the Cufflinks and the orthology annotations were merged into one track with the following steps (Fig. 1c): (i) cuffcompare (Trapnell *et al*. 2010) was used to compare the Cufflinks annotation with the orthology annotation, (ii) transcripts sharing the same junctions (cuffcompare category *=*) were merged with the orthology annotation by taking the longest 5′- and 3′-terminal exon between the Cufflinks and orthology annotations, (iii) transcripts corresponding to new isoforms (cuffcompare category *j*) were filtered by removing transcripts overlapping two different genes on the same strand using BEDTools (Quinlan & Hall 2010) (intersectBed – parameters -s -wb). The resulting new isoforms were incorporated into the final orthology annotation, (iv) transcripts not associated with any orthologues (cuffcompare category *i* and *u*, hereafter referred as putative novel transcripts) were separately retained for further processing. The same steps were used to merge the Trinity annotation to the combined annotation of orthology and Cufflinks (Fig. 1c).

### Annotation of novel genes

To annotate novel genes (i.e. those without any orthologues in *D. melanogaster*), we retained putative novel transcripts whose gene models shared the same junctions in both Cufflinks and Trinity (cuffcompare category *=*) and defined their 5′- and 3′-terminal exons by taking the longest exon from either the Cufflinks or Trinity annotation. Using BEDTools, we subsequently compared putative novel transcripts to those transcripts that were filtered out in the orthology annotation (see the section Orthology annotation). Putative novel transcripts not overlapping with the orthology set were defined as novel genes and named as dsim_PG00001, dsim_PG00002, etc. The overlapping ones were used to recover orthologues that had been missed due to overly stringent filtering: open reading frames (ORFs) were predicted for these transcripts using ORFPredictor (parameters strand = ‘+’), and protein sequences were aligned to the orthologoues proteins using BLASTP (Altschul *et al*. 1990). Genes were reassigned to the category ‘orthologue’ if the *D. simulans* and *D. melanogaster* proteins had a significant similarity (E < 10^−5^).

### Annotation of introns, CDS and UTRs

The exon annotation described above provided the introns coordinates as a byproduct. For each new isoform, the corresponding transcript sequence was extracted and processed with ORFPredictor (parameters strand = ‘+’) to find the corresponding CDS. Transcript-based coordinates of CDS were converted to genome coordinates using the script trcoord-to-genomecoord.py (Dryad ID doi:10.5061/dryad.ng95t). Finally, UTRs were defined for each transcript by the difference between the exon coordinates and the corresponding CDS coordinates. The final annotation (including exons, introns, CDS and UTRs) is provided in the standard GTF format (Dryad ID doi:10.5061/dryad.ng95t).

### Runs

Both DNA-Seq and RNA-Seq data sets were deposited in the NCBI Sequence Read Archive (SRA) under the Experiment ID SRX504933.

## Results and discussion

### Genome assembly and its evaluation relative to other *D. simulans* genome assemblies

We assembled ∼200 M reads in 5467 contigs with a N50 of 247 kb and an average coverage of 177x (Table 1). After scaffolding (see Materials and methods, section *De novo* assembly), the total length of the M252 genome is 121 196 329 bp, compared to 137 828 247 bp for FlyBase and 125 005 935 bp for Hu *et al*. (2012). These differences in length among the three assemblies are mainly due to the unassembled contigs of the FlyBase genome (Table 2). When considering only main chromosome arms, the genomes lengths are more similar: 109 695 738 bp for FlyBase, 118 453 741 bp for Hu *et al*. (2012) and 111 008 150 bp for M252. As the chromosomes were covered in the three assemblies to a different extent, depending on the amount of heterochromatin included, we compared orthologoues regions by extracting the largest syntenic regions from chromosomes 2L, 2R, 3L and 3R (Table S2, Supporting information). The X chromosome was not truncated because the synteny was conserved along the whole chromosome for all three references. The total length of the resulting truncated genomes for main chromosome arms is 109 088 562 bp for FlyBase, 112 646 407 bp for Hu *et al*. (2012) and 110 653 820 bp for the M252 assembly. The following analyses are based on these modified reference genomes.

**Table 1:** *De novo* assembly and scaffolding statistics for the *D. simulans* strain M252

Number of contigs	5467
N75	106 026
N50	246 600
N25	454 130
Minimum length	200
Maximum length	1 286 889
Average length	22 382
Total bp	122 360 288
Average coverage	177 X
Number of contigs without scaffold	3029
Median length of contigs without scaffold	557 bp

**Table 2:** Chromosome length and percentage of Ns for the three *D. simulans* assemblies

	FlyBase r1.4		Hu *et al*. (2012)		M252	
			
	Length (bp)	% Ns	Length (bp)	% Ns	Length (bp)	% Ns
2L	22 036 055	6.04	23 580 488	0.01	21 089 350	0.40
2R	19 596 830	7.08	21 589 432	0.01	18 979 895	0.13
3L	22 553 184	6.00	24 255 363	0.01	22 247 347	0.11
3R	27 517 382	5.64	27 160 941	0.01	26 970 900	0.07
4	949 497	14.91	1 026 345	0.03	1 104 516	0.56
X	17 042 790	15.33	20 841 172	0.02	20 616 142	0.44
Unassembled contigs	28 132 509	7.74	6 552 194	5.94	10 188 179	4.10

The percentage of Ns in the genome is substantially lower in the M252 and Hu *et al*. (2012) assemblies, compared to the FlyBase reference (Table 2), with the assembly of Hu *et al*. (2012) having the lowest percentage of Ns. The FlyBase reference contains more Ns as it might have many low coverage regions.

**Fig 2:**
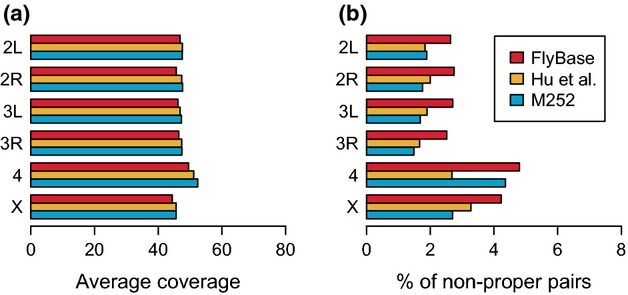
Coverage and percentage of nonproper pairs for the mapping of an independent *D. simulans* sample from Africa (Nolte *et al*. 2013) against the three *D. simulans* assemblies. Only statistics for main chromosome arms are shown.

To evaluate the quality of the assemblies, we mapped reads from an independent African *D. simulans* sample (see Materials and methods, section ‘Assembly evaluation’) to the three assemblies and computed the average coverage in windows of 10 kb for the main chromosome arms. The average coverage is similar among the different assemblies, but for most chromosome arms the M252 genome has a slightly higher coverage (Fig. 2a), suggesting a higher overall quality of the M252 assembly.

The occurrence of nonproper pairs is an indicator of low quality, as their presence is related to misassemblies in the reference. Initially, we calculated the average percentage of nonproper pairs on the main chromosome arms (Fig. 2b). The percentage of nonproper pairs is similar between the Hu *et al*. (2012) and M252 assemblies for both 2nd and 3rd chromosomes, while the 4th chromosome has an overall higher percentage of nonproper pairs in the M252 assembly compared to Hu *et al*. (2012). For a more detailed explanation of this discrepancy, see the Appendix s1 (Supporting information). Finally, the X chromosome shows the lowest percentage of nonproper pairs in the M252 assembly compared to the other two assemblies, suggesting that this chromosome has the highest overall quality in the M252 genome. Overall, the M252 and Hu *et al*. (2012) assemblies have a substantially lower percentage of nonproper pairs compared to the FlyBase assembly.

**Fig 3:**
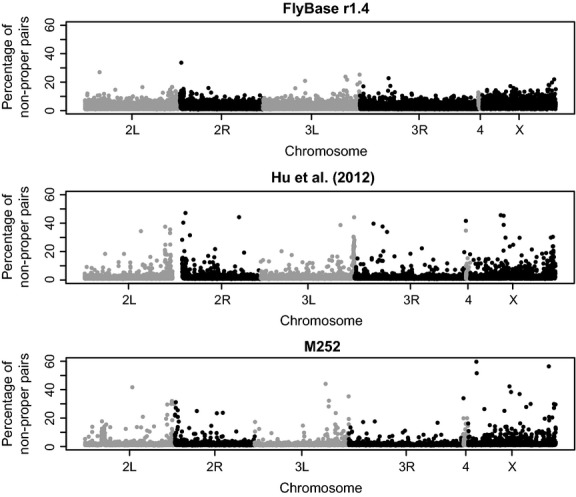
Distribution of nonproper pairs along each chromosomal arm in windows of 10 kb calculated after mapping an independent *D. simulans* African sample to the three assemblies.

To investigate the location of misassemblies, we plotted the percentage of nonproper pairs in windows of 10 kb on the main chromosomal arms (Fig. 3). The FlyBase reference shows a homogeneous distribution of nonproper pairs along the chromosomes, with a median of 2.5% per window. The Hu *et al*. (2012) and M252 assemblies show a more heterogeneous distribution of nonproper pairs, although the median percentage of nonproper pairs is lower compared to the FlyBase assembly. The overall high amount of nonproper pairs in the FlyBase reference might be explained by the fact that this genome was built by assembling a mixture of different strains. The spikes of broken pairs in the Hu *et al*. (2012) and the M252 assemblies are most likely the result of transposable elements or other repetitive structures that are underrepresented in the assemblies. The distribution of nonproper pairs along the chromosomes follows two patterns in the Hu *et al*. (2012) and the M252 assemblies: (i) an excess of nonproper pairs towards the centromeres, likely due to problems in assembling heterochromatic regions, (ii) a higher number of regions with nonproper pairs on the X chromosome compared to autosomes, which probably reflects a higher amount of repetitive sequences on the X (Gallach *et al*. 2007). Taken together, these results show that some regions are still intrinsically difficult to assemble with NGS data.

The use of an African sample could introduce a bias into the above evaluation as the w501 strain is most likely of North American origin (Begun *et al*. 2007), whereas the M252 strain is from Madagascar, and African and non-African *D. simulans* populations are known to be differentiated (Hamblin & Veuille 1999; Baudry *et al*. 2006). To avoid a bias due to sample origin, we repeated the assembly evaluation analysis using an independent sample from Europe and found comparable results (Fig. S1, Supporting information), suggesting that our conclusions are due to differences in assembly quality, rather than divergence between the read samples and the assemblies.

### Synteny analysis

**Fig 4:**
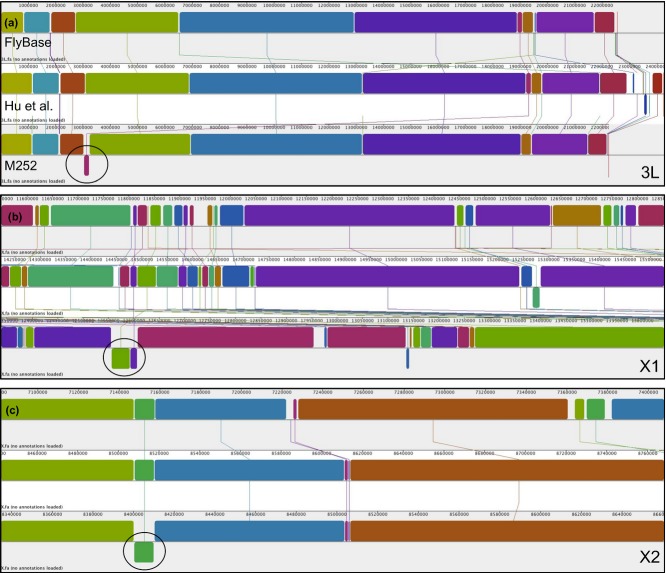
Synteny alignment of the three *D. simulans* assemblies highlighting with a circle the genomic rearrangements specific to the M252 assembly: a) Rearrangement 3L, b) Rearrangement X1, c) Rearrangement X2. Blocks of conserved synteny are shown with the same colour and connected by lines among the three assemblies. Blocks drawn below each horizontal line represent inverted regions.

Based on a full chromosome alignment generated with Mauve (Darling *et al*. 2004), we found that the synteny is well conserved along chromosome arms for all three genomes. At the centromere, synteny blocks are shorter and some rearrangements are present (Fig. 4a). Three major rearrangements specific to our assembly are apparent at locations 3L:3036720-3234344 (rearrangement 3L, Fig. 4a), X:8400345-8410149 (rearrangement X1, Fig. 4b) and X:12555584–12607284 (rearrangement X2, Fig. 4C). Rearrangements 3L and X1 appear to be inverted translocations, while rearrangement X2 looks like an inversion when compared to the other assemblies. To scrutinize the assembly for these regions, we aligned the initial reads of the M252 strain to the corresponding assembly and looked whether reads span rearrangement breakpoints in the M252 assembly. Support from paired-end reads confirmed the rearrangement 3L (Fig. S2A, B, Supporting information) and both rearrangements X1 (Fig. S3A, B, Supporting information) and X2 (Fig. S4A, B, Supporting information). The left side of both X1 and X2 rearrangements contained a stretch of Ns, which prevented mapping of paired-end reads. While these results indicate that these rearrangements are not assembly artefacts, we were interested to what extent our data support the arrangement in the other two assemblies. We mapped the M252 reads to the assembly of Hu *et al*. (2012) and evaluated the coverage at the breakpoints of the corresponding syntenic regions in that assembly. For rearrangements 3L (Fig. S2C, D, Supporting information) and X1 (Fig. S3C, D, Supporting information), we find support for at least one side of the alternative configuration, which suggests that we may have identified a segmental duplication that remained unnoticed in the *de novo* assembly, likely due to collapsed repetitive regions. Interestingly, we also find support for the alternative rearrangement X2 (Fig. S4C, D, Supporting information). Closer inspection, however, showed the presence of a repetitive stretch of poly-A at the end breakpoint of rearrangement X2 for both the inverted and the noninverted region (Fig. S5). This prevents a conclusive characterization of the correct configuration in M252 for rearrangement X2.

### Genome annotation

**Fig 5:**
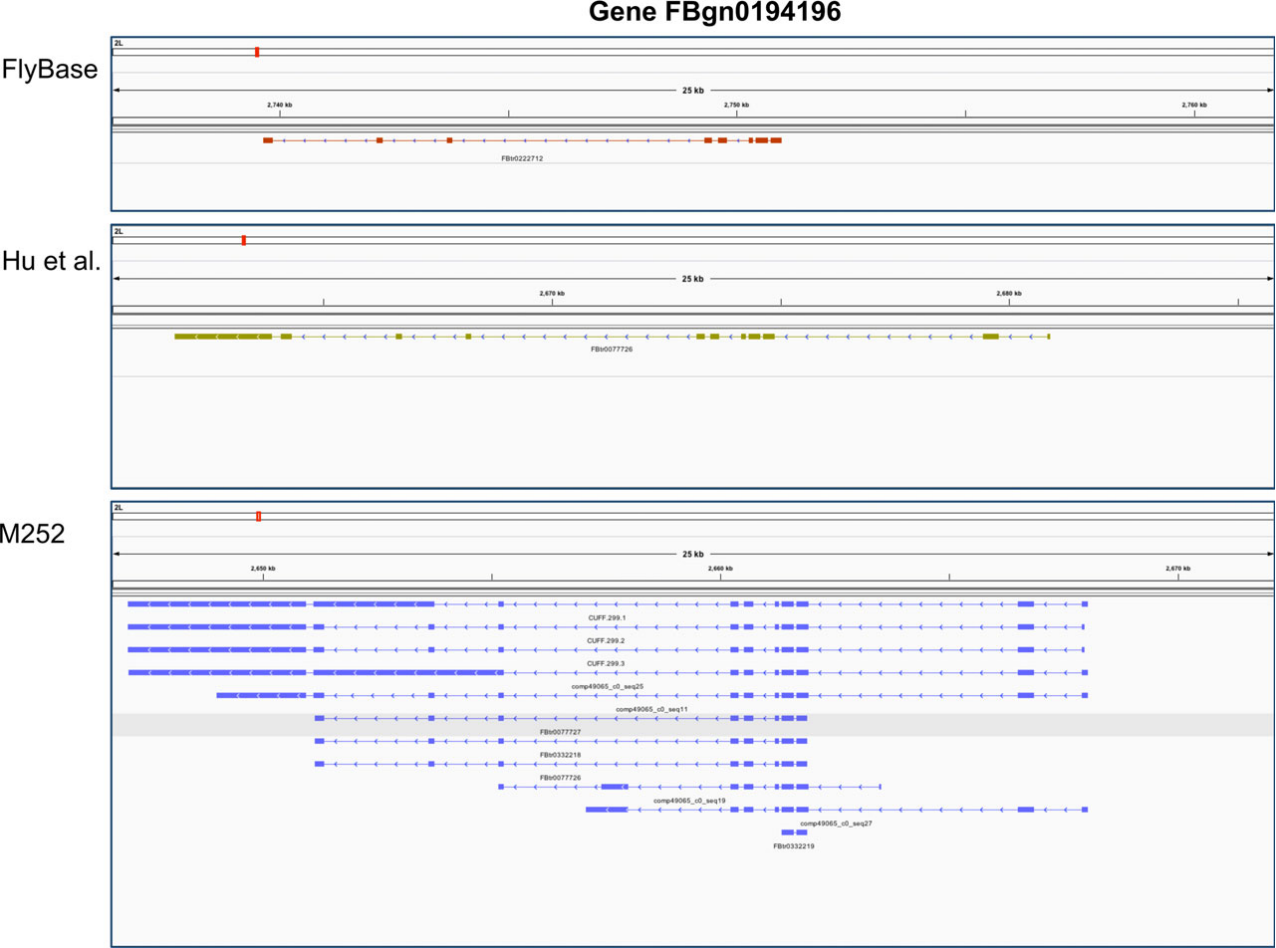
Comparison of the annotation at the gene locus FBgn0194196 among the three *D. simulans* assemblies. The M252 annotation contains many alternative transcripts, novel exons and longer UTRs compared to the other two assemblies.

We annotated protein-coding genes by merging orthology predictions from *D. melanogaster* with an annotation constructed using the RNA-Seq data set derived from a pool of multiple developmental stages (see Materials and methods, section ‘Library preparation’) and compared our annotation with the ones from FlyBase r1.4 and Hu *et al*. (2012) (Table 3). Among the 13968 genes in the *D. melanogaster* annotation from FlyBase r5.49, 12 974 (92%) had a corresponding orthologue in the M252 assembly based on stringent criteria (see Materials and methods, section Orthology annotation). We revealed abundant alternative splicing, by discovering a total of 9722 new isoforms, with an average of 2.5 isoforms per gene. In contrast, the FlyBase and the Hu *et al*. (2012) annotations have only a single isoform per gene (Table 3). In Fig. 5, we show the annotation for a representative locus for the three assemblies. Applying a series of conservative filtering steps, we identified 223 novel genes without a *D. melanogaster* orthologue, which are associated with 228 different isoforms. Using ORFPredictor, we predicted complete open reading frames (ORFs) for 8599 (88%) of the new isoforms and 156 (68%) of the isoforms associated to novel genes. Furthermore, we annotated UTRs for more than 70% of all the genes. By identifying ORF-disrupting mutations confirmed by multiple evidence (see Materials and methods, section Orthology annotation), we annotated for the first time a set of 781 putative pseudogenes. The FlyBase and Hu *et al*. (2012) assemblies contain two and zero pseudogenes, respectively. This set represents a good list of candidates for future studies on gene gains/losses and gene family evolution. In conclusion, our annotation constitutes a substantial improvement compared to the FlyBase and Hu *et al*. (2012) annotations, by revealing alternative splicing for 52% of the multiple-exon genes, UTRs for 70% of the genes and 223 novel genes.

**Table 3:** Comparison of genome annotations for the three different *D. simulans* assemblies

Genes	FlyBase r1.4	Hu *et al*. (2012)	M252
Total number of genes	15 548	10 786	12 990
Total number of genes + strand	7836	5416	6486
Total number of genes – strand	7712	5370	6504
Mean gene length	3.3 kb	3.8 kb	5.3 kb
Gene density genes/Mb	113	86	104
Number of transcripts	15 550	10 786	28 682
Average isoforms per gene	1.0	1.0	2.5
Percentage of transcripts with introns	77%	84%	90%
Mean transcript length	1.2 kb	2.0 kb	2.5 kb
Exons*			
Number	53 445	44 029	52 755
Mean number per transcript	3.4	4.1	4.1
GC content	52.9	46.5	50.4
Mean length (bp)	356	498	533
Total length (bp)	19 040 408	21 918 667	28 112 055
Introns*			
Number	37 897	33 222	39 767
Mean number per transcript	3.2	3.7	3.9
GC content	37.3	48.4	36.5
Mean length (bp)	870	585	887
Total length (bp)	32 971 902	19 446 332	35 255 589
UTRs*			
Number of genes with UTRs	4	9996	9274
Mean UTR length (bp)	2	262	455
Number of 5′UTRs	4	9549	8991
Mean 5′UTR length (bp)	2	206	367
Number of 3′UTRs	0	9786	8833
Mean 3′UTR length (bp)	–	317	543
Pseudogenes	2	0	781

* Statistics for transcripts, exons and UTRs refer to the longest isoform for each gene.

## Data Accessibility

*Sequence files*: DNA-Seq data set and RNA-Seq data set on NCBI SRA - ID SRX504933.

*Genome assembly and annotation on Dryad* - ID: doi:10.5061/dryad.ng95t - it contains the release of the assembly and annotation described in this manuscript.

*Genome assembly on NCBI* - ID: JMCE00000000 - it contains a new release of the assembly modified according to the NCBI policies.

*Script files on Dryad* - ID: doi:10.5061/dryad.ng95t.

## Supporting Information

**Fig. S1** Coverage and percentage of nonproper pairs for the mapping of an independent *D. simulans* sample from Europe (Portugal) against the three *D. simulans* assemblies. Only statistics for main chromosome arms are shown.

**Fig. S2** Support from paired-reads alignments (proper pairs only) at the breakpoints of rearrangement X2 in the M252 assembly (A, B) and at the corresponding synteny block in the Hu *et al*. assembly (C, D). The two dashed vertical lines indicate the position of the breakpoint. Reads marked in blue have a higher than expected insert-size. Only proper pairs with mapping quality >20 are shown.

**Fig. S3** Support from paired-reads alignments (proper pairs only) at the breakpoints of rearrangement X2 in the M252 assembly (A, B) and at the corresponding synteny block in the Hu *et al*. assembly (C, D). The two dashed vertical lines indicate the position of the breakpoint. Reads marked in red have a lower than expected insert-size, while reads marked in blue have a higher than expected insert-size. Only proper pairs with mapping quality > 20 are shown.

**Fig. S4** Support from paired-reads alignments (proper pairs only) at the breakpoints of rearrangement X2 in the M252 assembly (A, B) and at the corresponding synteny block in the Hu *et al*. assembly (C, D). The two dashed vertical lines indicate the position of the breakpoint. Reads marked in red have a lower than expected insert-size, while reads marked in blue have a higher than expected insert-size. Only proper pairs with mapping quality >20 are shown.

**Fig. S5** – A) Local alignment around the right-side coordinate of rearrangement X2 (100bp flanking, see Fig. S4B, D, Supporting information) between the Hu *et al*. and the M252 assemblies. The breakpoint corresponds to position 109 in the alignment. A repetitive sequence in the form of a poly-A microsatellite is present on the connection between the two synteny blocks.

**Table S1** Trimming statistics for the *D. simulans* strain M252. **Table S2** Coordinates of the largest common syntenic regions for the three assemblies.

**Appendix S1** Characterization of misassemblies on the 4th chromosome.
