# Supplementary figures and images for: Genome assembly and annotation of a Drosophila simulans strain from Madagascar
Source: Mol Ecol Resour. 2014 Jul 14;15(2):372–81. doi: 10.1111/1755-0998.12297 (PMC4344813; doi:10.1111/1755-0998.12297)

**A**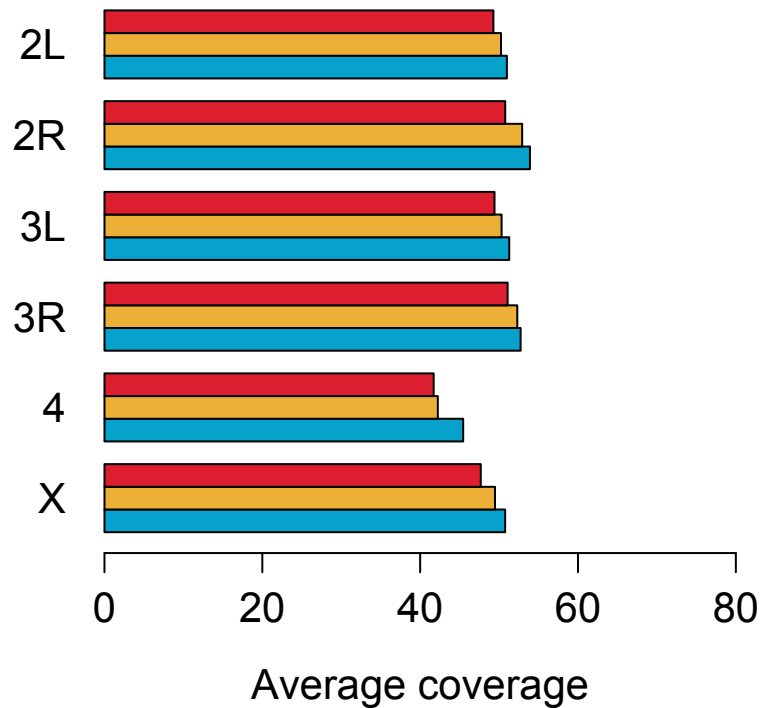**B**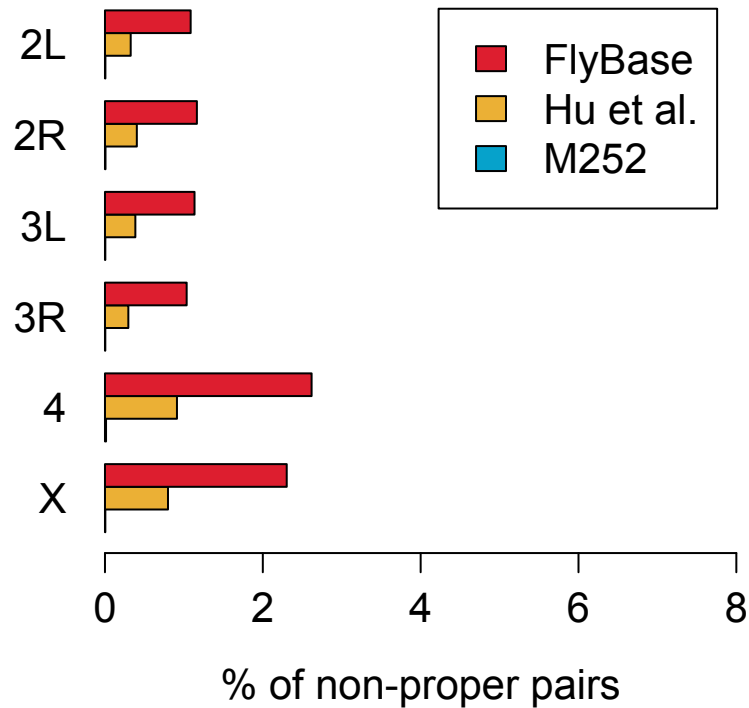

Supplement: Supplementary file 1 — Fig. S1 Coverage and percentage of nonproper pairs for the mapping of an independent D. simulans sample from Europe (Portugal) against the three D. simulans assemblies. Only statistics for main chromosome arms are shown. [file men0015-0372-sd1.pdf]

A

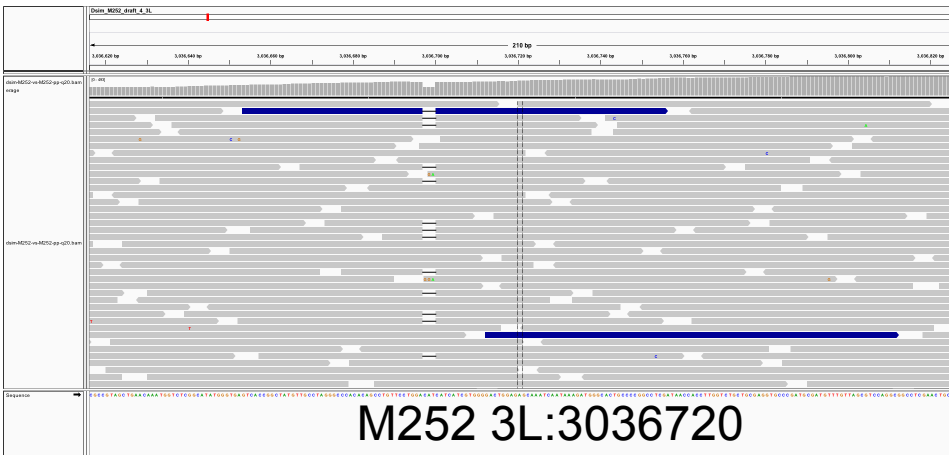

B

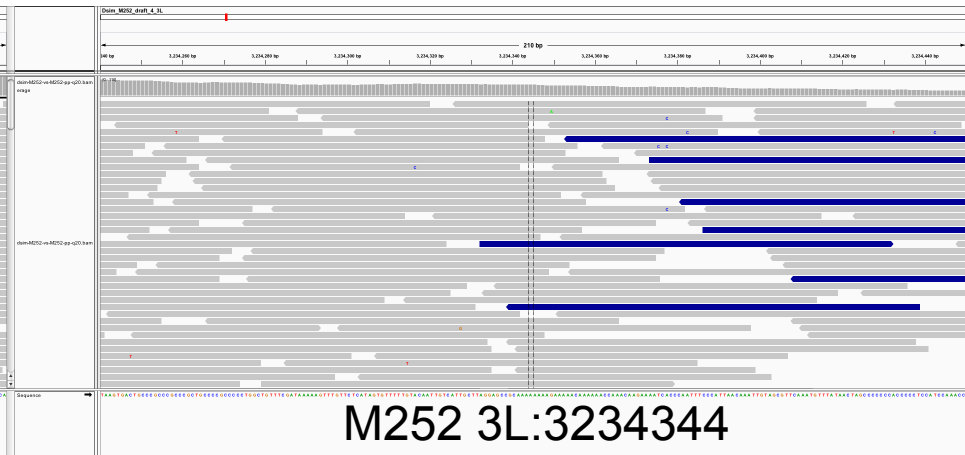

C

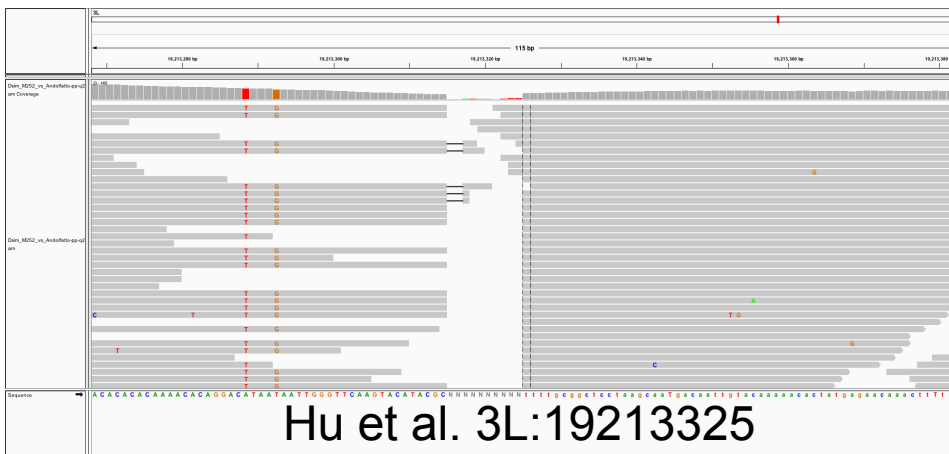

D

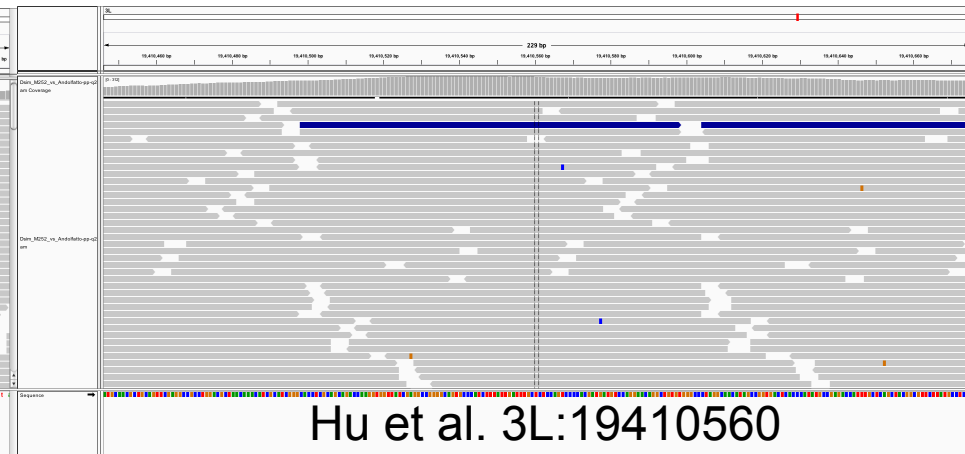

Supplement: Supplementary file 2 — Fig. S2 Support from paired-reads alignments (proper pairs only) at the breakpoints of rearrangement X2 in the M252 assembly (A, B) and at the corresponding synteny block in the Hu et al. assembly (C, D). The two dashed vertical lines indicate the position of the breakpoint. Reads marked in blue have a higher than expected insert-size. Only proper pairs with mapping quality >20 are shown. [file men0015-0372-sd2.pdf]

A

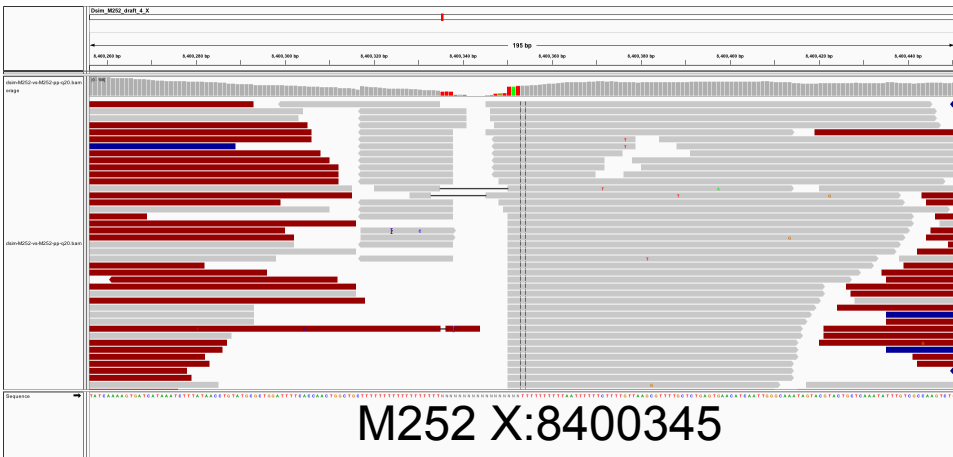

B

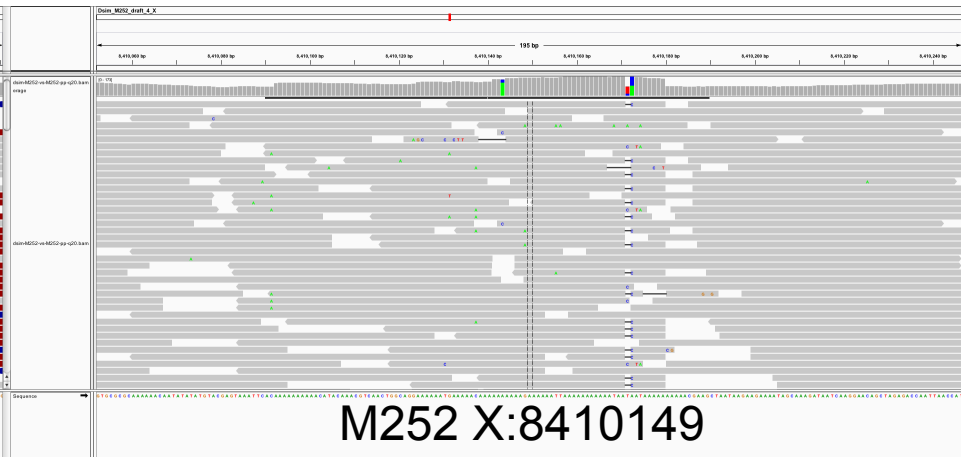

C

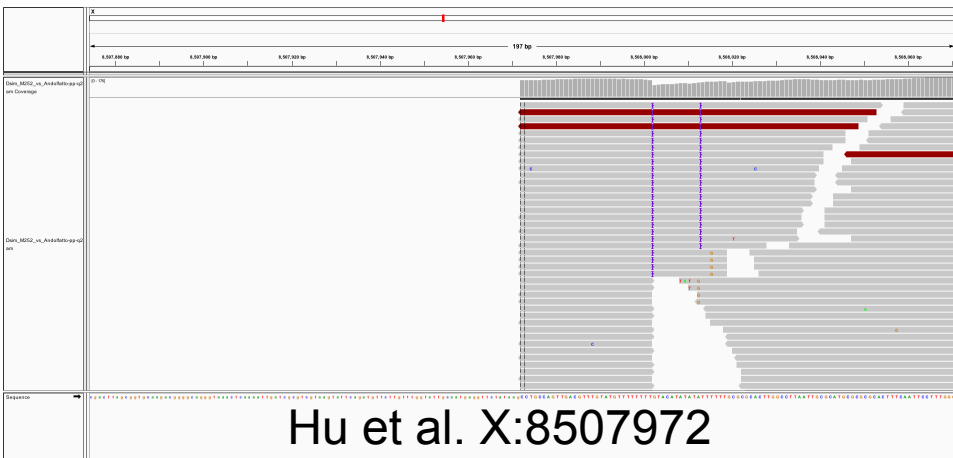

D

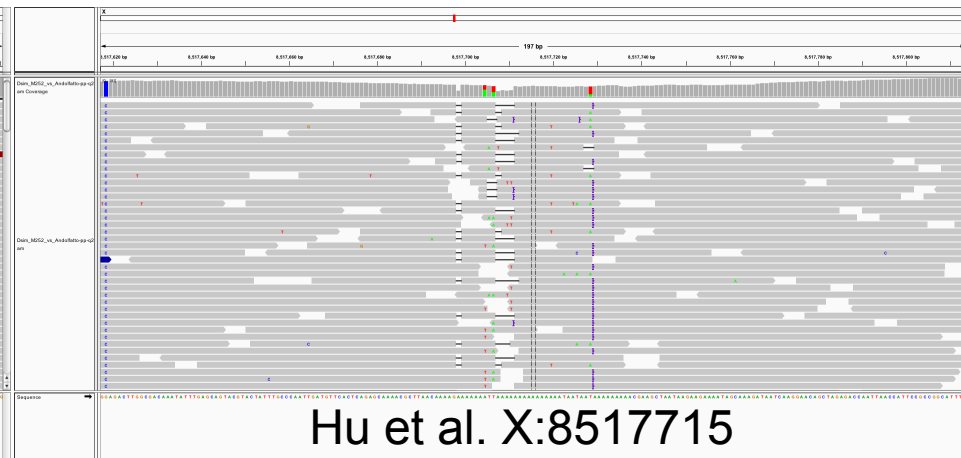

Supplement: Supplementary file 4 — Fig. S4 Support from paired-reads alignments (proper pairs only) at the breakpoints of rearrangement X2 in the M252 assembly (A, B) and at the corresponding synteny block in the Hu et al. assembly (C, D). The two dashed vertical lines indicate the position of the breakpoint. Reads marked in red have a lower than expected insert-size, while reads marked in blue have a higher than expected insert-size. Only proper pairs with mapping quality >20 are shown. [file men0015-0372-sd4.pdf]

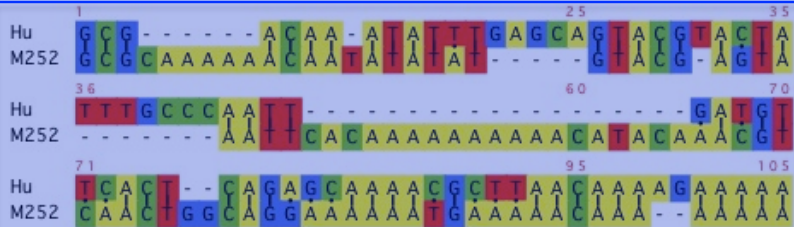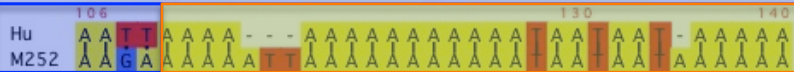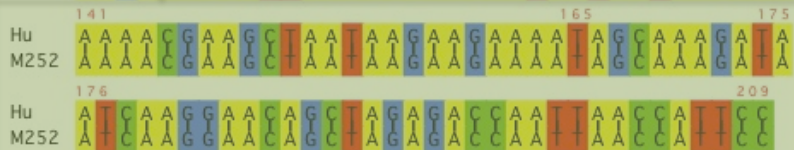

Supplement: Supplementary file 5 — Fig. S5 – A) Local alignment around the right-side coordinate of rearrangement X2 (100bp flanking, see Fig. S4B, D, Supporting information) between the Hu et al. and the M252 assemblies. The breakpoint corresponds to position 109 in the alignment. A repetitive sequence in the form of a poly-A microsatellite is present on the connection between the two synteny blocks. [file men0015-0372-sd5.pdf]
