# Supplementary material for: Genome assembly and annotation of a Drosophila simulans strain from Madagascar
Source: Mol Ecol Resour. 2014 Jul 14;15(2):372–81. doi: 10.1111/1755-0998.12297 (PMC4344813; doi:10.1111/1755-0998.12297)
Supplement: Supplementary file 3 — Fig. S3 Support from paired-reads alignments (proper pairs only) at the breakpoints of rearrangement X2 in the M252 assembly (A, B) and at the corresponding synteny block in the Hu et al. assembly (C, D). The two dashed vertical lines indicate the position of the breakpoint. Reads marked in red have a lower than expected insert-size, while reads marked in blue have a higher than expected insert-size. Only proper pairs with mapping quality > 20 are shown. [file men0015-0372-sd3.pdf]

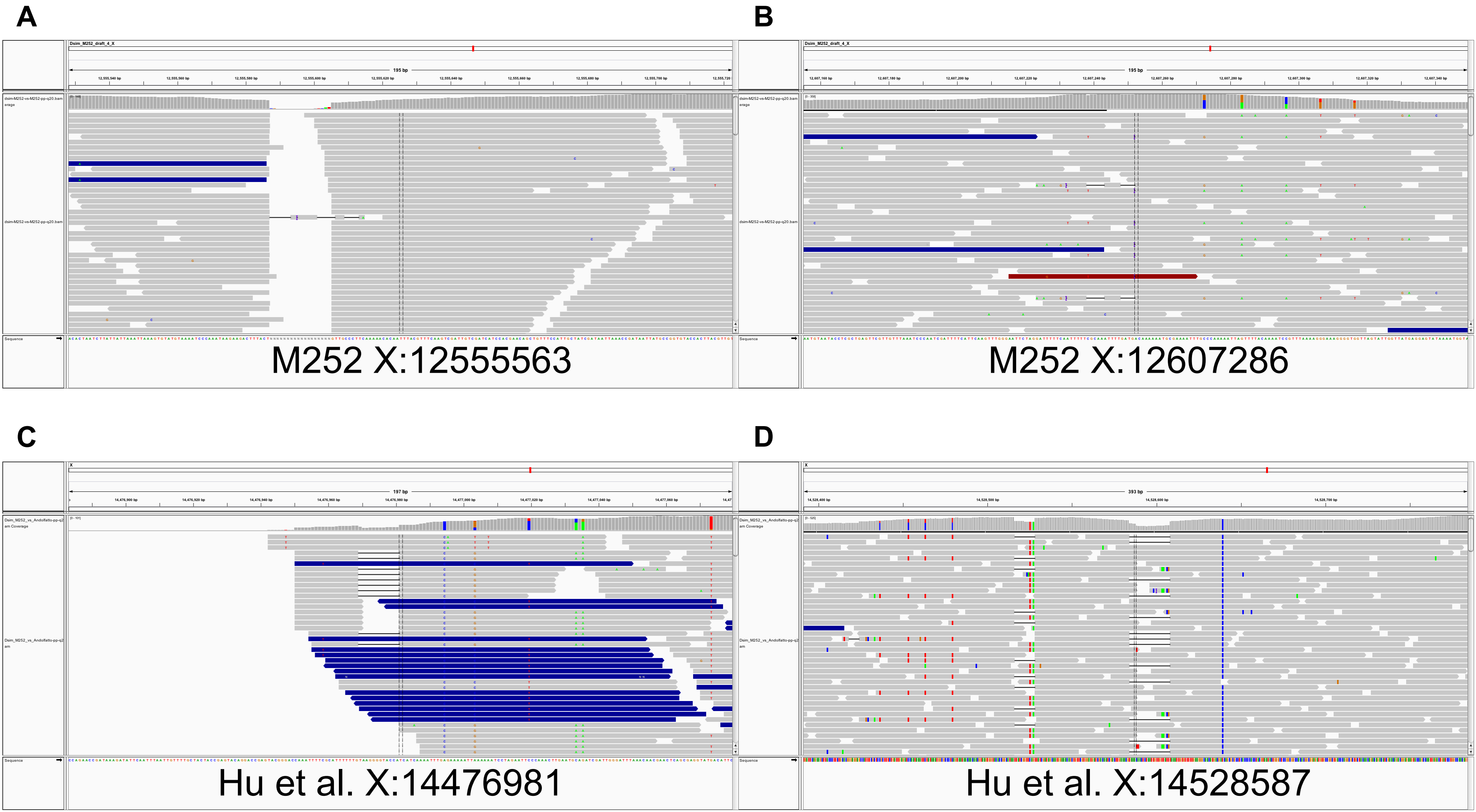
A
195 bp
M252 X:12555563
B
195 bp
M252 X:12607286
C
197 bp
Hu et al. X:14476981
D
393 bp
Hu et al. X:14528587
