# Supplementary material for: Genome assembly and annotation of a Drosophila simulans strain from Madagascar
Source: Mol Ecol Resour. 2014 Jul 14;15(2):372–81. doi: 10.1111/1755-0998.12297 (PMC4344813; doi:10.1111/1755-0998.12297)
Supplement: Supplementary file 6 [file men0015-0372-sd6.docx]

**Supplementary Tables**

**Supplementary Table 1** – Trimming statistics for the *D. simulans* strain M252

|  | DNA-Seq dataset | RNA-Seq dataset |
| --- | --- | --- |
| Total pairs | 200,332,558 | 184,756,175 |
| Trimmed pairs | 192,129,560 | 177,494,809 |
| Mean length | 97 bp | 97 bp |

**Supplementary Table 2** – Coordinates of the largest common syntenic regions for the three assemblies.

| **Chromosome** | **FlyBase r1.4** | **Hu et al. (2012)** | **M252** |
| --- | --- | --- | --- |
| 2L | 1 – 21781117 | 1 – 21453183 | 1 – 20976035 |
| 2R | 286970 – 19596830 | 2335415 – 21589432 | 167255 – 18979895 |
| 3L | 1 – 22487915 | 1 – 22910748 | 1 – 22173586 |
| 3R | 1 – 27517382 | 1 – 27160941 | 1 – 26970900 |
