# Supplementary material for: Genome assembly and annotation of a Drosophila simulans strain from Madagascar
Source: Mol Ecol Resour. 2014 Jul 14;15(2):372–81. doi: 10.1111/1755-0998.12297 (PMC4344813; doi:10.1111/1755-0998.12297)
Supplement: Supplementary file 7 — Appendix S1 Characterization of misassemblies on the 4th chromosome. [file men0015-0372-sd7.docx]

**Supplementary Text**

We investigated the excess of non-proper pairs on the on the 4^th^ chromosome and identified two regions with a peak of non-proper pairs specific for the M252 assembly at locations 4:532320- 532820 (region 4A) and 4:858577-858756 (region 4B). The higher coverage of region 4A compared to the flanking regions suggests a collapsed repeat. To validate this hypothesis we BLASTed region 4A to M252 and Hu et al. (2012) assemblies and detected a single hit on the 4^th^ chromosome for the M252 assembly and multiple best hits on the 4^th^, 2R and 3L chromosomes for the Hu et al. (2012) assembly, indicating that region 4A is a collapsed repeat in the M252 assembly. The location of region 4A in *D. melanogaster* also supports the configuration of the Hu et al. (2012) assembly. Region 4B is flanked by Ns and belongs to a single contig. BLAST analysis of this region against the M252 and Hu et al. (2012) assemblies revealed multiple best hits on different chromosomes for both assemblies, indicating that region 4B is an interspersed repeat that is difficult to place correctly.
